# Supplementary material for: Network mechanisms of ongoing brain activity’s influence on conscious visual perception
Source: Nat Commun. 2024 Jul 8;15:5720. doi: 10.1038/s41467-024-50102-9 (PMC11231278; doi:10.1038/s41467-024-50102-9)
Supplement: Supplementary file 1 — Supplementary Information [file 41467_2024_50102_MOESM1_ESM.pdf]

Network mechanisms of ongoing brain activity's influence on conscious visual perception

Yuan-hao Wu*, Ella Podvalny*, Max Levinson, Biyu J. He

*equal contributions

Supplementary information

Figure S1-7

Table S1-4

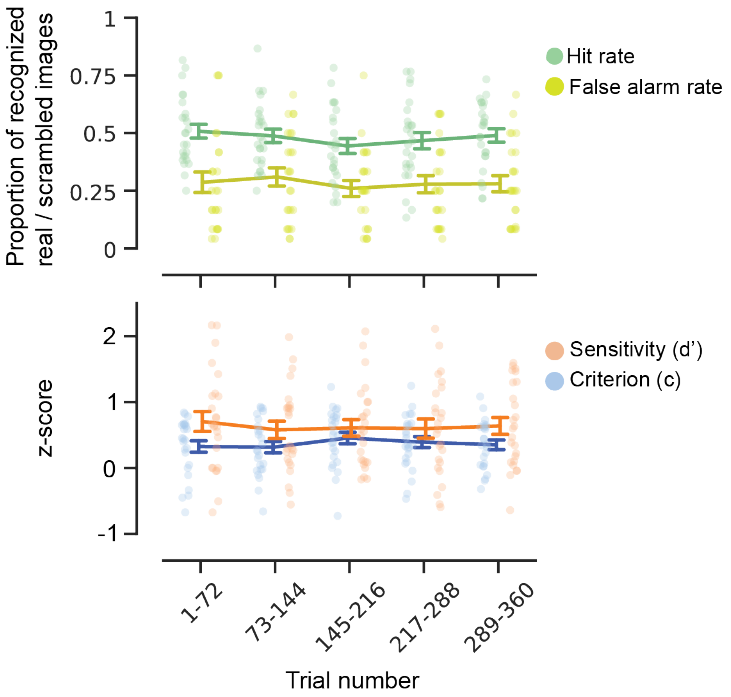

Figure S1. Perceptual behavior across the experiment, divided into five time bins. The line plots show the mean hit rate, false alarm rate, criterion and sensitivity across participants (*n* = 25) plotted as a function of trial groups sorted according to their presentation order. Circles represent individual participants. Error bars represent standard errors of the means (SEM). Separate two-sided 1x5 repeated-measures analysis of variance was conducted for each behavioral variable of interest. No significant effect of presentation order was detected at $p < 0.05$ uncorrected (Hit rate: $F_{4,96} = 1.68$, $p = 0.16$, $\eta^2 = 0.02$; False alarm rate: $F_{4,96} = 0.42$, $p = 0.79$, $\eta^2 = 0.01$; Criterion (c): $F_{4,96} = 1.18$, $p = 0.32$, $\eta^2 = 0.01$; Sensitivity (d'): $F_{4,96} = 0.32$, $p = 0.86$, $\eta^2 = 0.01$).

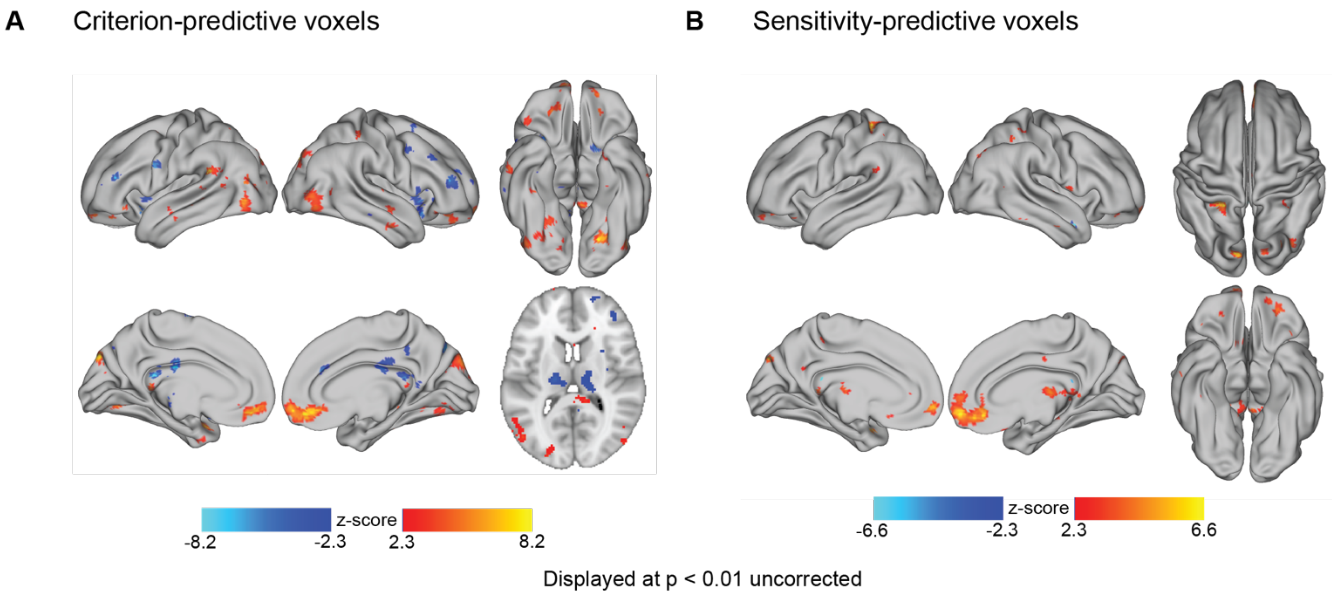

Fig S2 Voxels showing significant criterion- and sensitivity-predictive prestimulus activity (uncorrected). A Whole brain statistical maps for prestimulus activity's effects on criterion. Statistically significant positive and negative voxels are shown in warm and cool colors, respectively (n = 25, linear mixed-effects model, thresholded at p < 0.01, uncorrected). **B** Same as A, but for sensitivity.

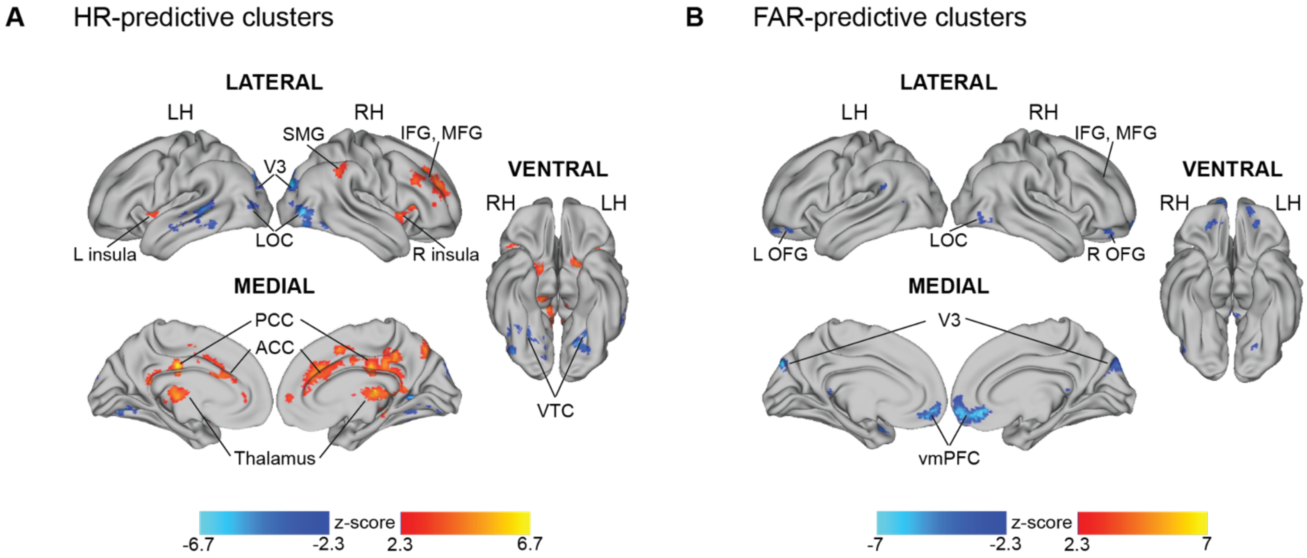

Fig S3 Anatomical sources of HR- and FAR-predictive prestimulus activity. A Whole brain statistical maps for prestimulus activity's influence on hit rates (HR). Statistically significant positive and negative voxel clusters are shown in warm and cool colors, respectively (n = 25, linear mixed-effects model, thresholded at p < 0.05, FWE corrected for cluster size, with a cluster-defining threshold (CDT) of p < 0.01). **B** Same as A, but for false alarm rate (FAR).

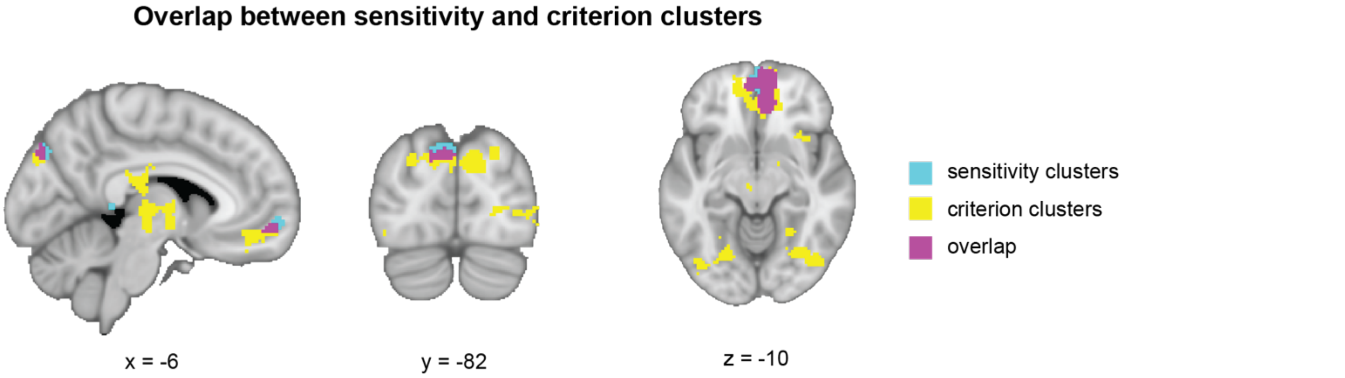

Fig S4 The unique and shared brain areas by sensitivity and criterion-predictive clusters. Sensitivity-predictive clusters as identified in the main analysis are displayed in blue, criterion-predictive clusters in yellow. Magenta depicts the areas shared by both sensitivity- and criterion-predictive clusters.

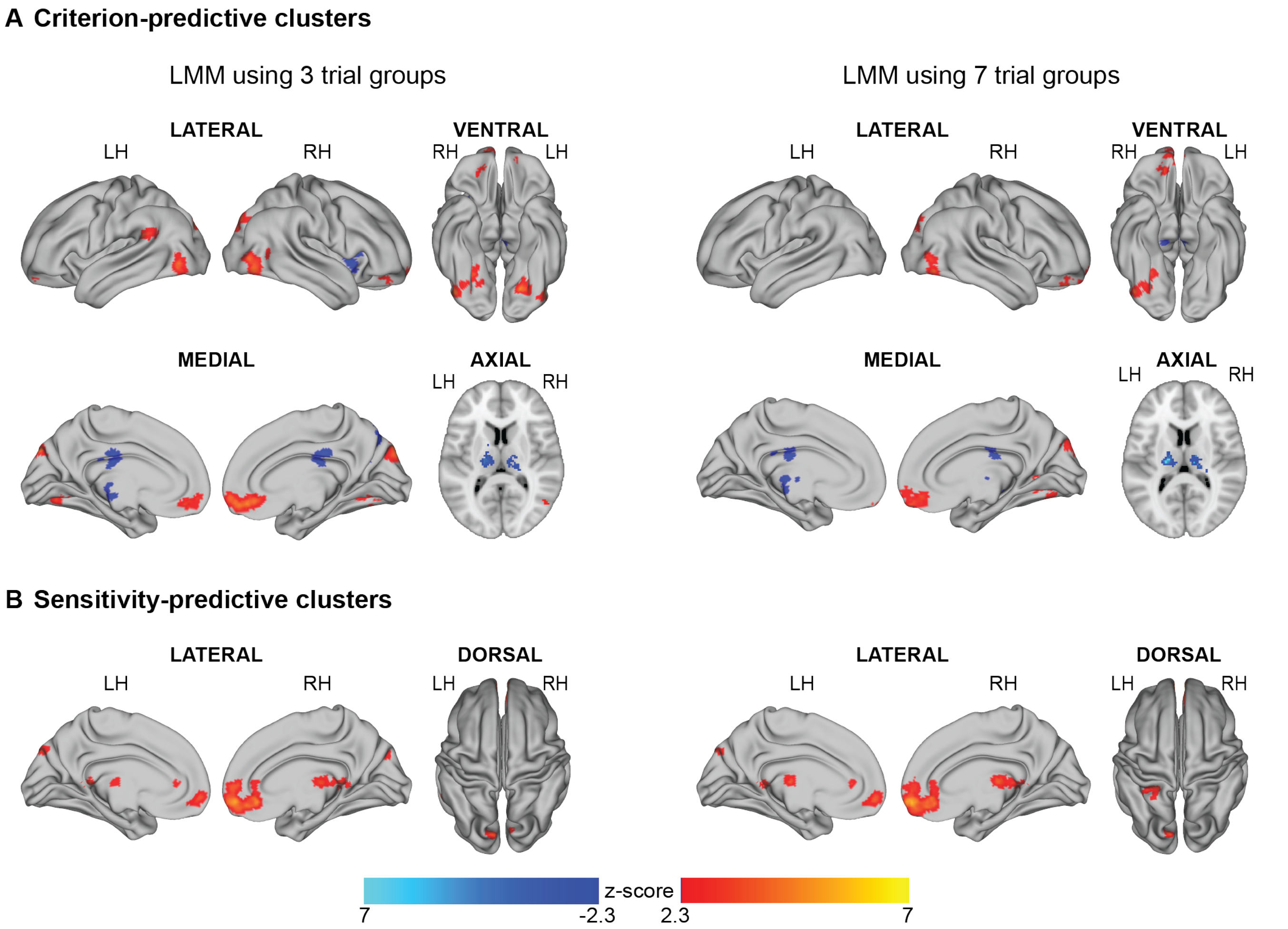

Fig S5 Criterion and sensitivity-predictive prestimulus activity clusters based on linear mixed-effects model (LMM) using 3 and 7 trial groups. A Whole brain statistical maps of prestimulus activity's influence on criterion based on LMM using 3 and 7 trial groups, respectively. Statistically significant positive and negative clusters are shown in warm and cool colors, respectively (n = 25, thresholded at $p < 0.05$, FWE corrected for cluster size, CDT at $p < 0.01$). **B** Same as A, but for sensitivity (d')-predictive prestimulus activity.

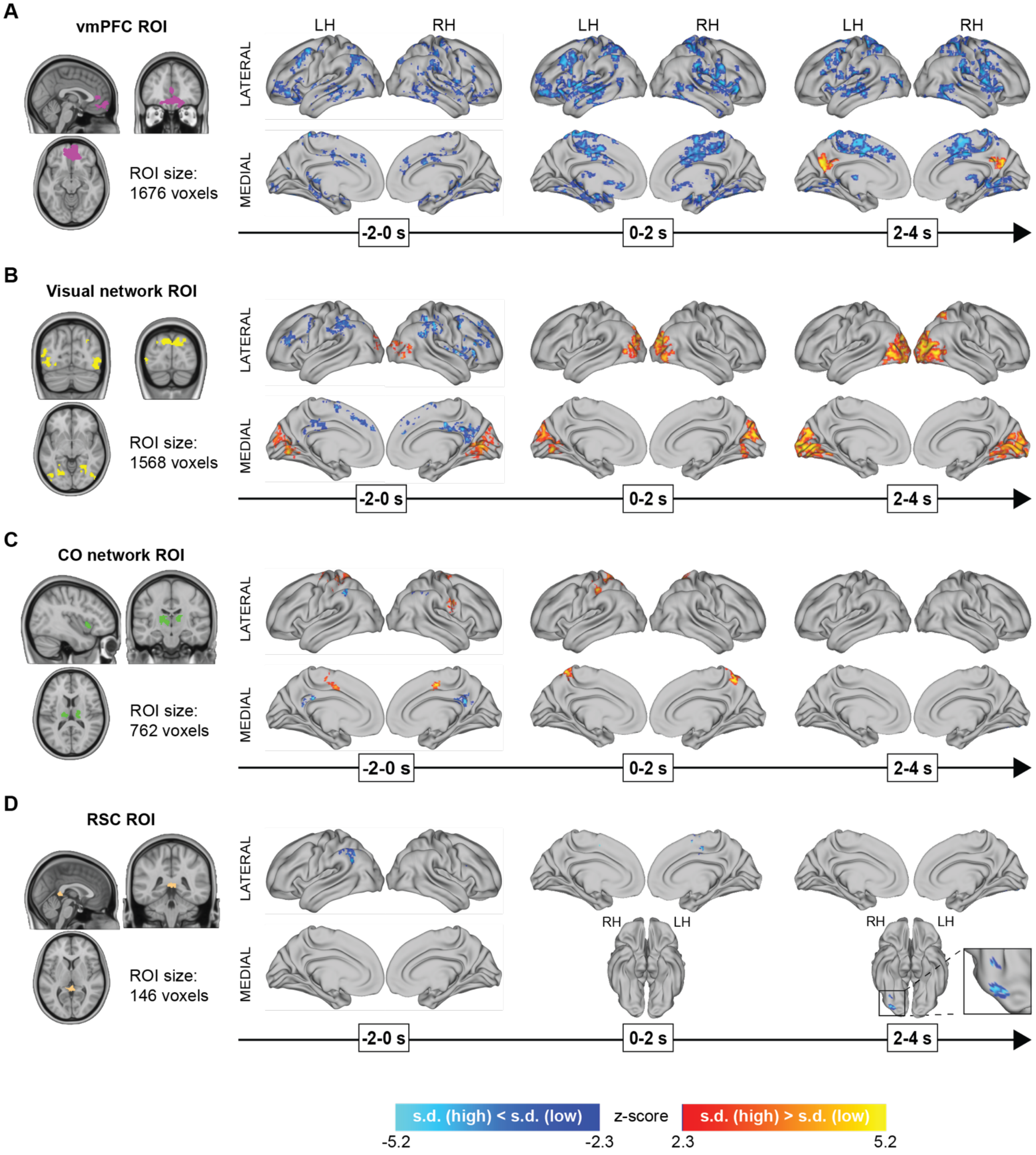

Fig S6 Prestimulus activity's influence on trial-to-trial variability of postimulus activity. A Left: vmPFC ROI. Right: Whole-brain statistical maps for differences in poststimulus variability between high and low vmPFC prestimulus activity trials from -2 to 4 s relative to the stimulus onset. Voxel clusters with significantly higher trial-to-trial variability (indexed by s.d.) in trials with high prestimulus activity are shown in warm colors, and voxel clusters with significantly lower response variability in trials with high prestimulus activity are shown in cool colors (n = 25, two-sided t-tests; thresholded at $p < 0.05$, FWE corrected for cluster size, CDT at $p < 0.01$). **B-D** Same as A, but for results obtained using visual network ROI (**B**), cingulo-opercular (CO) network ROI (**C**), and retrosplenial (RSC) ROI. (**D**). For vmPFC and visual network ROIs, results in the 0-2 s and 2-4 s windows (TR1 and TR2, respectively) are also presented in main text Fig 3.

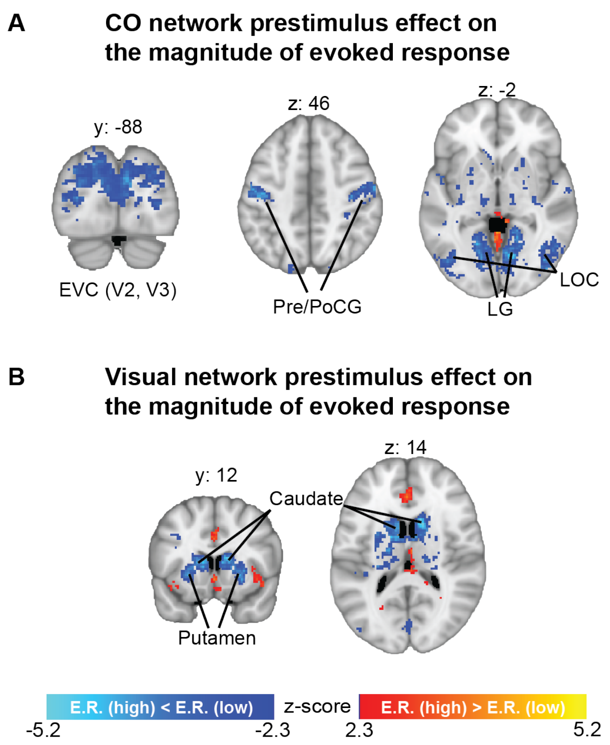

Fig S7 Prestimulus activity's influence on the magnitude of evoked response. A Whole-brain statistical maps for differences in the magnitude of evoked responses between high and low cingulo-opercular (CO) network prestimulus activity trials. Voxel clusters with significantly higher evoked responses (E.R.) under high CO network prestimulus activity are shown in warm colors, and voxel clusters with significantly lower evoked responses under high CO network prestimulus activity are shown in cool colors (two-sided t-tests; thresholded at $p < 0.05$, FWE corrected for cluster size, CDT at $p < 0.01$). **B** Same as A, but for results obtained using visual network prestimulus activity. EVC: early visual cortex; Pre/PoCG: pre/post-central gyrus; LG: lingual gyrus; LOC: lateral occipital cortex.

Table S1 Criterion-predictive clusters (*n* = 25, linear mixed-effects model). All results are reported at a cluster-corrected statistical level of $p < 0.05$ with an initial voxel-wise threshold of $p < 0.01$. Cluster size refers to the number of voxels in the cluster. MNI coordinates x, y, z and *z*-statistic refer to the peak voxels in each cluster. The clusters are listed in order of largest cluster size, separately for clusters showing positive and negative effects. In Table S1–S3, "Same cluster" means it belongs to the same cluster as the one listed in the previous row.

Brain regions	Cluster size	z-statistic	x	y	z
Positive clusters					
Ventromedial prefrontal cortex	1203	8.16	10	58	-10
R Lateral Occipital Cortex	831	5.15	42	-78	0
Bilateral V3 (Cuneal Cortex)	611	5.14	-4	-84	36
L Ventral Temporal Cortex (LG & FG)	423	5.03	-20	-70	-6
L Cerebellum	217	3.85	-24	-66	-34
L Lateral Occipital Cortex	177	3.92	-50	-72	8
L Angular Gyrus and Supramarginal Gyrus	174	5.96	-62	-52	20
Negative clusters					
L Thalamus	736	4.75	-18	-18	10
Anterior Posterior Cingulate Cortex	Same cluster	3.83	2	-14	28
R Thalamus	290	3.41	18	-26	12
R Anterior Insula	234	3.73	28	18	-14

FG: fusiform gyrus, LG: lingual gyrus. L: left, R: right.

Table S2 Same as Table S1, but for sensitivity-predictive regions.

Brain regions	Cluster size	z-statistic	x	y	z
Positive clusters					
Ventromedial Prefrontal Cortex	1045	6.58	10	58	-10
Retrosplenial Cortex	322	3.97	-2	-42	4
R Thalamus	Same cluster	3.79	2	-20	8
Bilateral V3 (Cuneal Cortex)	148	4.84	-6	-82	36
L Postcentral Gyrus	87	4.1	-24	-38	62
L Superior Parietal Lobule	Same cluster	3.91	-28	-38	60
R Lateral Occipital Cortex	66	3.67	28	-74	36

Table S3 Same as Table S1, but for HR-predictive regions.

Brain regions	Cluster size	z-statistic	x	y	z
Positive clusters					
Anterior Cingulate Cortex	4628	6.68	6	36	24
R anterior Insula	Same cluster	6.57	-40	-6	-6
R Thalamus	Same cluster	6.4	4	-22	10
L Thalamus	Same cluster	5.45	-8	-20	6
Frontal Pole	1031	5.44	32	46	24
Precuneus	568	4.91	2	-72	48
L Lateral Occipital Cortex	368	5.51	-24	-84	24
Brainstem	324	4.06	16	-28	-10
R Supramarginal Gyrus	300	3.74	54	-44	34
R Angular Gyrus	Same cluster	3.33	56	-46	52
L Middle Temporal Gyrus	259	4.81	-44	-18	-14
Negative clusters					
R Lateral Occipital Cortex	973	5.35	42	-76	-2
L Temporal Gyrus	467	4.73	-62	-44	-14
R V3 (Occipital Pole)	309	4.99	22	-88	28
R Lateral Occipital Cortex	Same cluster	3.59	16	-86	34
L VTC (FG and LG)	302	5.6	-32	-66	-4

FG: fusiform gyrus, LG: lingual gyrus. L: left, R: right.

Table S4 Same as Table S1, but for FAR-predictive regions.

Brain regions	Cluster size	z-statistic	x	y	z
Negative clusters					
Ventromedial Prefrontal Cortex	1292	7.02	10	58	-10
Bilateral V3 (Cuneal Cortex)	408	5.79	-6	-82	38
L Lateral Occipital Cortex	119	3.55	-50	-74	14
L Temporal Pole	116	-4.47	-32	6	-26
R Lateral Occipital Cortex	110	3.3	38	-78	2
L Angular Gyrus	99	-4.29	-64	-56	20
Ventral Temporal Cortex (FG)	99	4.46	-28	-80	-18
Retrosplenial Cortex	98	3.25	-2	-40	6

FG: fusiform gyrus. L: left, R: right.
