# Supplementary material for: Network mechanisms of ongoing brain activity’s influence on conscious visual perception
Source: Nat Commun. 2024 Jul 8;15:5720. doi: 10.1038/s41467-024-50102-9 (PMC11231278; doi:10.1038/s41467-024-50102-9)
Supplement: Supplementary file 3 — Reporting Summary [file 41467_2024_50102_MOESM3_ESM.pdf]

nature portfolio

Corresponding author(s): Biyu Jade He

Last updated by author(s): Jun 5, 2024

Reporting Summary

Nature Portfolio wishes to improve the reproducibility of the work that we publish. This form provides structure for consistency and transparency in reporting. For further information on Nature Portfolio policies, see our Editorial Policies and the Editorial Policy Checklist.

Statistics

For all statistical analyses, confirm that the following items are present in the figure legend, table legend, main text, or Methods section.

n/a	Confirmed	
☐	☒	The exact sample size (*n*) for each experimental group/condition, given as a discrete number and unit of measurement
☐	☒	A statement on whether measurements were taken from distinct samples or whether the same sample was measured repeatedly
☐	☒	The statistical test(s) used AND whether they are one- or two-sided<br>*Only common tests should be described solely by name; describe more complex techniques in the Methods section.*
☐	☒	A description of all covariates tested
☐	☒	A description of any assumptions or corrections, such as tests of normality and adjustment for multiple comparisons
☐	☒	A full description of the statistical parameters including central tendency (e.g. means) or other basic estimates (e.g. regression coefficient) AND variation (e.g. standard deviation) or associated estimates of uncertainty (e.g. confidence intervals)
☐	☒	For null hypothesis testing, the test statistic (e.g. *F*, *t*, *r*) with confidence intervals, effect sizes, degrees of freedom and *P* value noted<br>*Give P values as exact values whenever suitable.*
☒	☐	For Bayesian analysis, information on the choice of priors and Markov chain Monte Carlo settings
☒	☐	For hierarchical and complex designs, identification of the appropriate level for tests and full reporting of outcomes
☒	☐	Estimates of effect sizes (e.g. Cohen's *d*, Pearson's *r*), indicating how they were calculated

Our web collection on statistics for biologists contains articles on many of the points above.

Software and code

Policy information about availability of computer code

Data collection	Behavioral data collection was performed using MATLAB (R2017a). Stimuli were presented using the Psychophysics Toolbox (version 3) in MATLAB (R2017a).
Data analysis	Behavioral data were analyzed using Python 3.8. fMRI data were analyzed using FSL (version 5.0.10), Python packages including Nibabel (3.2.1), Nilearn (0.9.2), and scikit-learn (1.3.0) as well custom-written codes for Python . Code supporting this study is available at Code supporting this study is available at https://github.com/BiyuHeLab/NatCommun_Wu2024

For manuscripts utilizing custom algorithms or software that are central to the research but not yet described in published literature, software must be made available to editors and reviewers. We strongly encourage code deposition in a community repository (e.g. GitHub). See the Nature Portfolio guidelines for submitting code & software for further information.

Data

Policy information about availability of data

All manuscripts must include a data availability statement. This statement should provide the following information, where applicable:

- Accession codes, unique identifiers, or web links for publicly available datasets
- A description of any restrictions on data availability
- For clinical datasets or third party data, please ensure that the statement adheres to our policy

This paper consists of analysis of previously published data. Unthresholded whole-brain statistical maps generated in this study are available at https://neurovault.org/collections/17373/. Source data are provided with the paper.

Research involving human participants, their data, or biological material

Policy information about studies with human participants or human data. See also policy information about sex, gender (identity/presentation), and sexual orientation and race, ethnicity and racism.

Reporting on sex and gender	The classification into female and male was determined based on self-report. No gender-based analyses were performed as there were no gender-specific hypotheses regarding the neural processing associated with the visual object recognition. The findings do not apply to specific sexes or genders.
Reporting on race, ethnicity, or other socially relevant groupings	The findings do not apply to specific races, ethnicities, or other socially relevant groupings
Population characteristics	see below in Research sample section
Recruitment	Participants were recruited via flyers, e-mail, and an online research participant recruit platform (researchmatch.org). Since these are common ways of subject recruitment for human neuroscience subjects, we do not expect any resulting potential self-selection bias.
Ethics oversight	fMRI experiment was approved by the Institutional Review Board of New York University School of Medicine (protocol #15-01323).

Note that full information on the approval of the study protocol must also be provided in the manuscript.

Field-specific reporting

Please select the one below that is the best fit for your research. If you are not sure, read the appropriate sections before making your selection.

☐ Life sciences ☒ Behavioural & social sciences ☐ Ecological, evolutionary & environmental sciences

For a reference copy of the document with all sections, see nature.com/documents/nr-reporting-summary-flat.pdf

Behavioural & social sciences study design

All studies must disclose on these points even when the disclosure is negative.

Study description	Quantitative experimental study including recordings of visual perceptual behavior and functional magnetic resonance imaging (fMRI) data in healthy adult volunteers.
Research sample	The research sample consisted of healthy human volunteers living in New York City (n=38, 26 females, mean age 27.2, range 20 to 38), including students and non-students. Sample is representative due to an age range covering young adults and both males and females. Rationale for subject selection included availability and sufficiently high level of task performance.
Sampling strategy	A convenience sample was recruited through volunteer subject pools at New York University. We chose a sample size similar to those used in recent published fMRI studies on perception and cognition conducted with healthy human volunteers, such as:<br><br>Carlos González-García, Matthew W. Flounders, Raymond Chang, Alexis T. Baria, and Biyu J. He. Content-specific activity in frontoparietal and default-mode networks during prior-guided visual perception. eLife. 2018 Jul; 7:e36068.
Data collection	The participants were alone inside the fMRI room and experimenters monitored in an adjacent room. Behavioral data were collected via computer (MATLAB, Psychophysics Toolbox). fMRI data were recorded via 7T Siemens MRI scanner and 32-channel head coil. The researchers were not blind to the study hypotheses.
Timing	The data were collected from February 2018 - February 2019.
Data exclusions	1 block from 1 subject was excluded due to a scanner error.

Data exclusions	2 blocks (1 subject), 1 block (1 subject), and 4 blocks (1 subject) were excluded due to excessive motion artifacts in the fMRI data. Complete data from 3 participants were excluded due to poor performance in the main behavioral task. Exclusion criteria were established prior to the beginning of the study. For decoding analyses that needed training and testing set of trials, we required more than 5 trials in each set.
Non-participation	4 participants did not complete the experiment due to poor performance on a screening task. 6 participants declined to complete the experiment due to discomfort.
Randomization	Participants were not allocated to experimental groups, since all subjects were presented with the same stimuli (within-subject paradigm). Stimuli were presented in randomized order.

Reporting for specific materials, systems and methods

We require information from authors about some types of materials, experimental systems and methods used in many studies. Here, indicate whether each material, system or method listed is relevant to your study. If you are not sure if a list item applies to your research, read the appropriate section before selecting a response.

Materials & experimental systems

n/a	Involved in the study
☒	☐ Antibodies
☒	☐ Eukaryotic cell lines
☒	☐ Palaeontology and archaeology
☒	☐ Animals and other organisms
☒	☐ Clinical data
☒	☐ Dual use research of concern
☒	☐ Plants

Methods

n/a	Involved in the study
☒	☐ ChIP-seq
☒	☐ Flow cytometry
☐	☒ MRI-based neuroimaging

Plants

Seed stocks	*Report on the source of all seed stocks or other plant material used. If applicable, state the seed stock centre and catalogue number. If plant specimens were collected from the field, describe the collection location, date and sampling procedures.*
Novel plant genotypes	*Describe the methods by which all novel plant genotypes were produced. This includes those generated by transgenic approaches, gene editing, chemical/radiation-based mutagenesis and hybridization. For transgenic lines, describe the transformation method, the number of independent lines analyzed and the generation upon which experiments were performed. For gene-edited lines, describe the editor used, the endogenous sequence targeted for editing, the targeting guide RNA sequence (if applicable) and how the editor was applied.*
Authentication	*Describe any authentication procedures for each seed stock used or novel genotype generated. Describe any experiments used to assess the effect of a mutation and, where applicable, how potential secondary effects (e.g. second site T-DNA insertions, mosiacism, off-target gene editing) were examined.*

Magnetic resonance imaging

Experimental design

Design type	Task, event-related
Design specifications	15 blocks per subject. Each block includes 24 trials of 8-10 seconds each, with a 6-20 second inter-trial interval (jittered according to an exponential distribution).
Behavioral performance measures	Correct object categorization button press and subjective recognition report button press (yes or no). Task performance was considered acceptable if recognition rate for each category was above 15%, and if categorization accuracy for recognized images was at least 30% higher than for unrecognized images.

Acquisition

Imaging type(s)	Functional and structural MRI
Field strength	7 Tesla
Sequence & imaging parameters	Structural MPRAGE: gradient echo, 3D imaging, FOV 256 mm, matrix size 256x256, slice thickness 1mm, 192 sagittal slices, TE 4.49 ms, TR 3000 ms, flip angle 6 deg. Structural proton density: gradient echo, 3D imaging, FOV 256 mm, matrix size 256x256, slice thickness 1mm, 192 sagittal slices, TR 1760 ms, TE 2.57 ms, flip angle 6 deg. Functional: gradient echo, EPI, FOV 192 mm, matrix size 96x96, slice thickness 2mm (10% distance factor), oblique

orientation, TR 2000 ms, TE 25 ms, flip angle 50 deg, multiband factor 2, GRAPPA acceleration 2, phase encoding direction P -> A.

Area of acquisition: Whole brain

Diffusion MRI: ☐ Used ☒ Not used

Preprocessing

Preprocessing software	FSL version 5.0.10. Anatomical brain extraction using BET.<br>Functional preprocessing using FEAT. Brain extraction using BET, 3.0 mm and 4.0 mm FWHM smoothing kernel for task and localizer runs, high pass filtering with 150 second temporal cutoff, grand mean scaling, slice timing correction
Normalization	Linear boundary-based registration from functional to anatomical space. Linear registration (12 degrees of freedom) from anatomical to standard MNI152 space.
Normalization template	MNI152 group standard space
Noise and artifact removal	Artifact removal using independent component analysis (MELODIC): manually inspect 30-40 components that together explain ~75% of variance in the BOLD signal. Artifacts related to motion, arteries, or CSF pulsation were removed.<br>Correction for magnetic field inhomogeneity: the anatomical MPRAGE image was divided by the anatomical proton density image.
Volume censoring	3 parameters of head rotation and 3 parameters of head translation were derived using MCFLIRT in FSL version 5.0.10. Blocks containing excessive motion (>6mm spike in the relative mean displacement timecourse) were removed.

Statistical modeling & inference

Model type and settings	Voxel-wise residuals obtained from general linear models at the individual research subject level were used to approximate the prestimulus spontaneous activity.<br>Prestimulus activity's influence on perceptual behavior was assessed using linear mixed-effects models, included fixed effects for the trial group and intercept while treating participants as a random effect on the intercepts.<br>Prestimulus activity's influence on trial-to-trial variability of poststimulus activity was assessed using a model-free approach.<br>Prestimulus activity's influence on magnitude of poststimulus activity was assessed using general linear model: fixed effects for each task block, fixed effects for each subject across runs, mixed effects across subjects.<br>The signal detection theory simulation was carried out using linear regression models
Effect(s) tested	prestimulus effect vs null

Specify type of analysis: ☐ Whole brain ☐ ROI-based ☒ Both

Anatomical location(s)	Four ROIs: visual network, CO network, vmPFC, and retrosplenial cortex (RSC) were defined based on statistically significant criterion- and sensitivity-predictive clusters obtained from the linear mixed-effects models
Statistic type for inference<br>(See Eklund et al. 2016)	Cluster inference. Cluster-defining threshold of $p < 0.01$, cluster size threshold $p < 0.05$.
Correction	FWE cluster correction for whole brain analysis. FDR correction for ROI analysis.

Models & analysis

n/a	Involved in the study
☒	☐ Functional and/or effective connectivity
☒	☐ Graph analysis
☐	☒ Multivariate modeling or predictive analysis

Multivariate modeling and predictive analysis	Searchlight decoding was performed on subject-level data using logistic regression models (c = 1) implemented in scikit-learn. We employed a 6 mm radius spherical searchlight and moved it voxel-by-voxel through the entire brain. At each voxel location, the decoder was trained to distinguish between object categories based on the patterns of BOLD responses within the searchlight during the functional localizer scan. The decoder was subsequently applied to response patterns corresponding to a specific prestimulus activity level in the main task. The decoders' prediction performance was evaluated using balanced decoding accuracy. This process was performed for high and low prestimulus activity trials, respectively. Significant difference in decoding accuracy between high and low prestimulus activity trials at group level was assessed t-test, corrected for multiple comparisons at cluster level at $p < 0.05$.
